# Clinical and Molecular Characteristics of Neonatal Extended-Spectrum β-Lactamase-Producing Gram-Negative Bacteremia: A 12-Year Case-Control-Control Study of a Referral Center in Taiwan

**DOI:** 10.1371/journal.pone.0159744

**Published:** 2016-08-09

**Authors:** Ming-Horng Tsai, I-Ta Lee, Shih-Ming Chu, Reyin Lien, Hsuan-Rong Huang, Ming-Chou Chiang, Ren-Huei Fu, Jen-Fu Hsu, Yhu-Chering Huang

**Affiliations:** 1 Division of Neonatology and Pediatric Hematology/Oncology, Department of Pediatrics, Chang Gung Memorial Hospital, Yunlin, Taiwan; 2 Division of Pediatric Neonatology, Chang Gung Memorial Hospital, Taoyuan, Taiwan; 3 Chang Gung University of Science and Technology, Chiayi, Taiwan; 4 Department of Anatomy, College of Medicine, China Medical University, Taichung, Taiwan; 5 Department of Pediatrics, Chang Gung Memorial Hospital, Taoyuan, Taiwan; 6 College of Medicine, Chang Gung University, Taoyuan, Taiwan; 7 Division of Pediatric Infectious Disease; Chang Gung Memorial Hospital, Taoyuan, Taiwan; Beijing Institute of Microbiology and Epidemiology, CHINA

## Abstract

Extended-spectrum β-lactamase (ESBL)-producing Gram-negative bacteremia (GNB) in the neonatal intensive care unit was characterized by comparison with two control groups: a susceptible control group and a general base population group over 2001 to 2012. The influence of ESBL production on mortality was studied in all study subjects and ESBL-GNB isolates were microbiologically characterized. We identified 77 episodes of ESBL-GNB (14.2% of all neonatal late-onset GNB), which were caused by *Klebsiella* spp. (62.3%), *E*. *coli* (20.8%) and *Enterobacter* spp. (16.9%). Most ESBL-GNB strains were genetically unrelated and the SHV-type ESBLs were the most prevalent (67% of isolates). Comparison with both control groups disclosed previous usage of 3^rd^ generation cephalosporin (odds ratio [OR], 4.72; 95% confidence interval [CI], 2.03–10.97; P < 0.001), and underlying renal disease (OR, 4.07; 95% CI, 1.10–15.08; P = 0.035) as independent risk factors for ESBL-GNB. Inadequate empiric antibiotics, a higher illness severity, higher rates of infectious complications and sepsis-attributable mortality were more frequently seen in neonates with ESBL-GNB than those with non-ESBL GNB (20.8% and 15.6% vs. 9.2% and 7.9%, respectively; *P* = 0.008 and 0.049, respectively). Neonates with underlying secondary hypertension (OR, 7.22; 95% CI, 2.17–24.06) and infectious complications after bacteremia (OR, 6.66; 95% CI, 1.81–19.31) were identified as independent risk factor for in-hospital mortality. ESBL-GNB accounted for one-seventh of all neonatal gram-negative bacteremia, especially in neonates exposed to broad-spectrum cephalosporins. Neonates with ESBL-GNB bacteremia more frequently received inadequate empirical antibiotic therapy, which were associated with a higher rate of infectious complications and an adverse outcome.

## Introduction

Gram-negative bacilli are the second common cause of bloodstream infection (BSI) in the neonatal intensive care unit (NICU) [1,2]. Extended-spectrum β-lactamase (ESBL)-producing Gram-negative bacilli are the leading cause of nosocomially acquired multidrug-resistant organisms [3,4] and have been responsible for an increasing number of NICU outbreaks [5–7]. Infections caused by β-lactamase-producing Enterobacteriaceae have serious implications on NICU infection control practices, and are often associated with a delay in effective antibiotics administration. Besides, treatment options for ESBL-producing Gram-negative bacteremia (ESBL-GNB) are often limited, since these microorganisms are often resistant to other antimicrobials such as aminoglycosides, trimethoprim/sulfamethoxazole, or quinolones [8].

Currently, most studies on ESBLs in the NICU focused on successful control or molecular epidemiology of single outbreak [6,7,9–11]. Some investigated risk factors for colonization, acquisition and infection, or emergence of antimicrobial resistance [11–16]. Little is known about the clinical features about ESBL-GNB in the NICU. Moreover, these studies were limited by a suboptimal control group selection, comparing case patients with and without ESBL-producing pathogens infections [13–15], small sample size [15–18], or analyzing all nosocomial infections instead of focusing on bacteremia [5,13–15,18]. We therefore conducted this study to assess the clinical features, risk factors, and molecular epidemiology of ESBL-GNB in the NICU using a case-control-control analysis, which was recommended by Kaye *et al* [19] to overcome the limitations aroused by usual matched case-control studies.

## Materials and Methods

### Study setting, participants and study design

This study was carried out in the NICU of Chang Gung Memorial Hospital (CGMH), which contains three units with a total capacity of 49-bed in tertiary-care level and 58-bed of special care nurseries in a university-affiliated teaching hospital in Taiwan. The annual admissions were around 1,700 infants, and two-fifths of them were critically ill or preterm infants requiring mechanical ventilation. All infants < 34 to 35 weeks’ completed gestation, with a birth weight < 2 kg or > 5 kg, or with any clinical signs of respiratory distress or cardiovascular, gastrointestinal, or neurologic problems requiring surgical or intensive treatment were eligible to admission in our NICU. This study was approved by the institutional review board of Chang Gung Memorial Hospital, with a waiver of informed consent because all patient records/information were anonymized and de-identified prior to analysis.

From January 2001 to December 2012, all the episodes of ESBL-GNB BSI were included in this study. Because only *E*. *coli*, *K*. *pneumonia*, *K*. *oxytoca*, *E*. *cloacae*, and *E*. *aerogenes* were ever identified as the causative microorganisms of neonatal ESBL-GNB, all neonatal late-onset BSI episodes due to these five pathogens without producing ESBL during the same period constituted the control group A. The control group B was from all hospitalized neonates: three control patients per case patient were chosen from those who were admitted within half a month before or after the case patient in the same unit, and had a hospital stay longer than the age at onset of ESBL-GNB in case patients. Polymicrobial infection was included in the cases if one of the isolates was an ESBL-producing strain, and also included in the first control group if one of the isolates was a GNB. All episodes of late-onset BSI were considered as different independent events, and we considered a new episode of BSI when the same organism was identified after a 14-day course of appropriate antibiotic therapy or one or more negative blood culture, or if a different organism was identified from a subsequent culture 7 days after the first one.

### Definitions and variables

Criteria from the Centers for Disease Control (CDC) and Prevention were applied to define neonatal bacteremia or BSI [20]. Late-onset BSI was defined as at least one positive blood culture obtained after 72 hours of life. Congenital infection and early-ones sepsis were defined as a definite infectious focus and septicemia that occurred within the first 72 hours of life [3]. All comorbidities of prematurity, including respiratory distress syndrome (RDS), intraventricular hemorrhage (IVH), bronchopulmonary dysplasia (BPD), and necrotizing enterocolitis (NEC) were based on the latest updated diagnostic criteria in the standard textbook of neonatology [21].

Shock was defined as a mean blood pressure < lower limit according to gestational age that was unresponsive to fluid treatment or required vasoactive agents [22]. Empirical antibiotics were considered to be inappropriate if the treatment regimen did not include at least one antibiotic that was active in vitro against the infecting microorganisms within 24 hours of blood culture collection. Prior antibiotic therapy was defined as systemic antibiotic > 72 hours in the preceding 30 days before bacteremia onset. The outcomes variables were the in-hospital mortality, and we also compared the sepsis-attributable mortality and infectious complications between ESBL-GNB and the control group A. Infectious complications were defined as a newly infectious focus or persistent organ dysfunction which occurred within one week and directly related to bacteremia, but not concurrently at onset of bacteremia. Sepsis attributable mortality was defined as neonates who expired within three days after onset of bacteremia, those who died of infectious complications or clinically progressive deterioration since onset of bacteremia.

### Data collection

In addition to a prospectively collected database as previously described [23,24], medical records of all study subjects were reviewed to collect the following data: exposure to central venous catheter (CVC), total parenternal nutrition (TPN), antibiotic or other medication, use of mechanical ventilation, clinical courses of all episodes of bacteremia, and treatment outcomes. The illness severity was evaluated by neonatal therapeutic intervention scoring system (NTISS) [25], calculated at the most severe period during the whole BSI episode. In the case group and the control group A, categorical variables were identified at the onset of bacteremia and continuous variables were identified before the onset of bacteremia. For control group B, these variables were identified throughout the whole course of hospital stay.

### Microbiological Characterization

All blood samplings were ordered by the attending physicians in the presence of clinical features compatible with systemic inflammatory response syndrome or when infection was suspected. Blood cultures were obtained through peripheral venous puncture (never through a CVC) and then performed using the BACTEC 9240 system. Antibiotic susceptibility patterns were determined according to methods recommended by the National Committee for Clinical Laboratory Standards Institute (CLSI) for disk diffusion method and categorical assignment was carried out using CLSI breakpoints [26]. ESBL production was screened and confirmed in all isolates with a profile suggestive of resistance by performing a double-disc synergy test according to CLSI guidelines [27]. The presence of *bla*_SHV_, *bla*_DHA_, *bla*_CMY_ and *bla*_CTX-M_ genes was investigated by polymerase chain reaction (PCR) amplification, as previously described [28,29]. Molecular characterizations of ESBL-producing isolates were typed by infrequent-restriction-site PCR, and restriction patterns were analyzed by applying previously established criteria [29].

### Statistical analysis

Categorical variables were compared by using the χ^2^ test or the Fisher exact test, and Mann-Whitney *U* test and the *t* test were used for comparison of continuous variables, depending on the distribution. To investigate the independent risk factors of ESBL-GNB and final mortality, conditional logistic regression was used to compute crude odds ratios (ORs) and 95% confidence intervals (CIs). Variables with a crude *P* value of < 0.1, those that were biologically sound, and those found in previous studies of ESBL-producing Enterobacteriaceae were introduced into the multivariate analysis performed by conditional logistic regression and then a stepwise backward process. All statistical analyses were performed using the SPSS version 15.0 (IBM SPSS Statistics, IBM Corporation, Armonk, NY).

## Results

**Fig 1:**
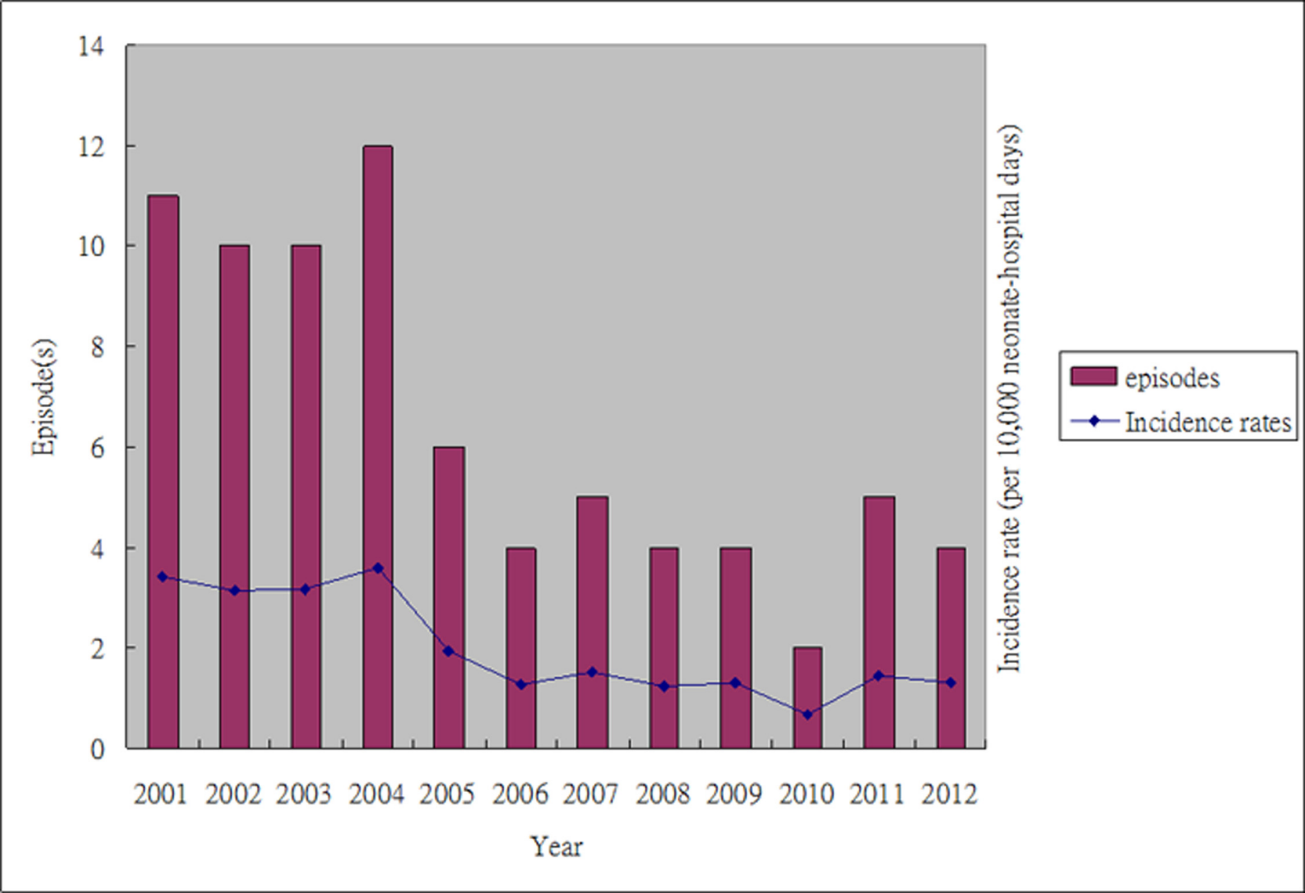
Bacteremia due to extended-spectrum β-lactamase (ESBL)-producing *Enterobacteriaceae* in the neonatal intensive care unit, 2001–2012, Episodes and clinical incidence rates per year.

During the study period, 542 episodes of Gram-negative bacteremia (GNB) were identified in the NICU of CGMH. Of them, 77 (14.2%) episodes occurred in a total of 71 patients were identified as ESBL-producing isolates, including *E*. *coli* (16 episodes), *K*. *pneumonia* (40 episodes), *K*. *oxytoca* (8 episodes), *E*. *cloacae* (12 episodes), and *E*. *aerogenes* (1 episode). All these ESBL-GNB were late-onset BSI. The control group A consisted of 316 episodes of GNB in 289 neonates, and the pathogens were *E*. *coli* (94 episodes), *K*. *pneumonia* (143 episodes), *K*. *oxytoca* (22 episodes), *E*. *cloacae* (37 episodes), and *E*. *aerogenes* (20 episodes). Only 8 patients had both ESBL and non-ESBL GNB during the study period. More than half (43/77, 55.8%) of ESBL-GNB occurred before December 31, 2004 (first third of the study period) (Fig 1), and the incidence rate of ESBL-GNB decreased significantly after January 2005 when compared with that before the end of 2004 (1.34 vs. 3.33 per 10,000 neonate-hospital days, p < 0.01).

### Risk factors for neonatal bacteremia due to ESBL-producing microorganisms

Demographics, perinatal history, most chronic comorbidities, and age at onset of BSI of the patients were comparable between the ESBL-GNB group and the non-ESBL GNB group. The duration of central venous catheter (CVC), ventilator and total parenternal nutrition (TPN) usages, and hospital stay were also comparable. Neonates with ESBL-GNB had a significantly higher rate of underlying congenital anomalies, gastrointestinal sequelae, renal disorders, and more frequent exposure to most antibiotics than those with non-ESBL GNB (Table 1). When compared with the control group B, which was made up of 231 uninfected patients, neonates with ESBL-GNB had higher rates of outborn, low Apgar score at 5 minutes, most chronic conditions, and more frequent exposure to antibiotics, CVCs placement, and TPN usage. Longer duration of CVC placement, ventilator and TPN uses, and hospital stay were also noted.

**Table 1:** Demographic and clinical characteristics of all study subjects, including 77 episodes of ESBL gram-negative bacteremia (GNB) [Case episodes], 316 episodes of non-ESBL GNB bacteremia (Control Group A), and 231 uninfected controls (Control Group B).

Variable	Case episodes (n = 77 episodes)	Control group A (n = 316 episodes)	Control group B (n = 231 patients)	*P* value*
Birth weight (g), median (IQR)	1580 (953–2510)	1445 (968–2450)	1455 (965–2320)	0.790, 0.258
Gestational age (weeks), median (IQR)	30.0 (27.0–37.0)	31.0 (27.0–35.8)	31.0 (27.0–36.0)	0.838, 0.428
Male gender	39 (50.6)	177 (56.0)	132 (57.1)	0.444, 0.355
Outborn	34 (44.1)	116 (36.7)	71 (30.7)	0.241, 0.037
Age at onset of bacteremia (day), median (IQR)	28.0 (16.0–64.5)	26.0 (14.0–50.0)	-	0.149, n/a
Isolates period				< 0.001, 1.00
2001–2004	43 (55.8)	98 (31.0)	129 (55.8)	
2005–2012	34 (44.2)	218 (69.0)	102 (44.2)	
Perinatal history				
Birth by cesarean section	47 (61.0)	169 (53.5)	156 (67.5)	0.350, 0.332
Low Apgar Score at 5 minutes (≤ 7)	32 (41.6)	118 (37.3)	66 (28.6)	0.515, 0.047
Prolonged rupture of membrane (PROM > 18 hours)	16 (20.8)	58 (18.4)	34 (14.7)	0.628, 0.216
Maternal fever or chorioamnionitis	6 (7.8)	16 (5.1)	7 (3.0)	0.404, 0.098
Congenital infection and/or early onset sepsis	4 (5.2)	12 (3.8)	0 (0)	0.529, 0.036
Underlying chronic condition^¶^				
Congenital anomalies^¥^	9 (11.7)	12 (3.8)	4 (1.7)	0.010, 0.001
Neurological sequelae, congenital or acquired	16 (20.8)	46 (14.6)	9 (3.9)	0.221, < 0.001
Cardiovascular disease^$^	7 (9.1)	15 (4.7)	18 (7.8)	0.223, 0.810
Bronchopulmonary dysplasia	11 (14.3)	91 (28.8)	61 (26.4)	0.009, 0.030
Pulmonary hypertension and/or cor pulmonale	1 (1.3)	4 (1.3)	9 (3.9)	0.982, 0.461
Congenital gastrointestinal tract pathology	7 (9.1)	23 (7.3)	5 (2.2)	0.632, 0.013
Gastrointestinal sequelae^**^	10 (13.0)	17 (5.4)	6 (2.6)	0.025, 0.001
Renal disease^&^	9 (11.7)	6 (1.9)	0 (0)	< 0.001, < 0.001
Surgical history (within one month) ^€^	8 (10.4)	26 (8.2)	16 (6.9)	0.505, 0.331
Use of corticosteroid (within one month) ^€^	6 (7.8)	24 (7.6)	20 (8.8)	0.953, 0.813
Invasive mechanical ventilation (intubation) ^€^	58 (75.3)	250 (79.1)	157 (68.0)	0.796, 0.343
On high frequency oscillatory ventilator^€^	7 (9.1)	17 (5.6)	30 (13.0)	0.223, 0.362
Use of TPN and/or intrafat^€^	70 (90.9)	269 (85.1)	182 (78.8)	0.267, 0.017
Use of central venous catheter^€^	73 (94.8)	282 (89.2)	186 (80.5)	0.195, 0.002
Antibiotic exposure (within 30 days before bacteremia)^€^				
3^rd^ generation cephalosporin	53 (68.8)	78 (24.7)	85 (36.8)	< 0.001, < 0.001
Vancomycin or Teicoplanin	34 (64.1)	65 (20.6)	72 (31.2)	< 0.001, 0.052
Carbapenem	12 (15.6)	21 (6.6)	10 (4.3)	0.020, 0.003
Monobactam	3 (3.9)	17 (5.4)	4 (1.7)	0.595, 0.270
Aminoglycoside	51 (66.2)	173 (54.7)	193 (83.5)	0.073, 0.002
Anti-fungal drugs	4 (5.2)	4 (1.3)	3 (1.3)	0.029, 0.068
Anti-anaerobes antibiotics (metronidazole)	14 (18.2)	12 (3.8)	6 (2.6)	< 0.001, < 0.001
Episode of bacteremia				0.125, n/a
First episode	60 (77.9)	269 (85.1)	-	
Recurrent episode	17 (22.1)	47 (14.9)	-	
Duration of TPN and/or intrafat^#^	31.0 (12.0–56.0)	25.0 (12.0–53.8)	11.0 (5.0–19.0)	0.510, < 0.001
Duration of ventilator use^#^	27.0 (9.0–61.0)	26.0 (7.3–63.0)	16.0 (4.0–46.0)	0.890, < 0.001
Duration of intubation^#^	20.0 (4.0–45.0)	17.0 (3.0–46.0)	6.0 (0–25.0)	0.124, < 0.001
Duration of central venous catheter use^#^	44.0 (25.0–68.0)	35.0 (20.0–63.5)	13.0 (5.0–24.0)	0.074, < 0.001
Duration of hospital stay^#^	71.0 (43.0–110)	64.0 (36.0–102)	45.0 (28.0–74.0)	0.433, < 0.001
Overall in-hospital mortality^#^	14/71 (19.7)	36/289 (12.5)	9/231 (3.9)	0.126, < 0.001

All data were expressed as number (percentage %), unless indicated otherwise; IQR: interquartile range; TPN: total parenternal nutrition; n/a: not available.

*P values were expressed as comparisons between (Case episodes and Control group A, Case episodes and Control group B).

^#^Data were 71 unique patients with ESBL GNB and 289 unique patients with non-ESBL GNB.

^¶^At onset of Gram-negative (both ESBL and non-ESBL) bacteremia, and during the total hospital stay for control group B, one patient may have more than one chronic conditions.

^€^One month before onset of Gram-negative (both ESBL and non-ESBL) bacteremia, and during the total hospital stay for control group B.

^¥^Included all documented and undocumented syndrome, chromosome anomalies, genetic or metabolic disorder, but not simple cleft palate or polydactyly.

^$^Including patients with complicated congenital heart disease and acyanotic heart disease with heart failure sign.

**Including short bowel syndrome, GI pseudo-obstruction, adhesion ileus, hepatic failure and chronic malnutrition.

^&^Including congenital nephrotic syndrome, chronic renal insufficiency, renal failure requiring hemodialysis and IgA nephropathy.

To identify independent risk factors for the occurrence of ESBL-GNB, the results of multivariate logistic regression are summarized in Table 2. Only two factors, antibiotic exposure to broad-spectrum cephalosporins and underlying renal disease were found independently associated with occurrence of ESBL-GNB when the case groups were compared to both the non-ESBL GNB and uninfected controls.

**Table 2:** Multivariate analysis of risk factors for neonatal late-onset bloodstream infection due to Extended-spectrum β-lactamase (ESBL)-producing Gram-negative bacilli (GNB).

Control group	non-ESBL GNB neonates population		base population of hospitalized neonates	
Risk factor	adjusted OR (95% CI)	*P* value	adjusted OR (95% CI)	*P* value
Outborn	-	-	2.23 (1.19–4.19)	0.013
Low apgar score at 5 minute (≤ 7)	-	-	0.84 (0.42–1.68)	0.616
Underlying chronic conditions^¶^				
Congenital anomalies	2.09 (0.64–6.83)	0.225	3.33 (0.73–15.24)	0.121
Neurological comorbidities	-	-	4.37 (1.52–12.58)	0.006
Congenital GI tract pathology	-	-	0.94 (0.20–4.50)	0.939
Gastrointestinal sequelae	1.55 (0.58–4.22)	0.394	2.38 (0.61–9.40)	0.213
Renal disease	5.18 (1.37–19.52)	0.015	4.07 (1.10–15.08)	0.035
Use of TPN and/or intrafat*	-	-	3.88 (0.62–24.48)	0.482
Use of central venous catheter*	-	-	0.57 (0.12–2.73)	0.148
Previous antibiotic exposure*	-	-		
3^rd^ generation cephalosporin	6.88 (3.53–13.38)	< 0.001	4.72 (2.03–10.97)	< 0.001
Vancomycin or teicoplanin	1.19 (0.60–2.37)	0.619	3.27 (1.36–7.87)	0.008
Aminoglycoside	3.13 (1.66–5.89)	< 0.001	-	-
Carbapenem	1.42 (0.53–3.80)	0.492	2.01 (0.65–6.27)	0.228
Anti-fungal drugs	0.97 (0.19–4.99)	0.968	1.80 (0.25–13.04)	0.560
Anti-anaerobes antibiotics (metronidazole)	2.06 (0.74–5.78)	0.169	5.91 (1.81–19.31)	0.003

^¶^At onset of Gram-negative (both ESBL and non-ESBL) bacteremia, and during the total hospital stay for control group B, one patient may have more than one chronic conditions, Each patient with an underlying chronic condition is compared with those without that specific condition.

*One month before onset of Gram-negative (both ESBL and non-ESBL) bacteremia, and during the total hospital stay for base population of hospitalized neonates (control group B).

ESBL: Extended-spectrum β-lactamase; GNB: Gram-negative bacilli; HFOV: high frequency oscillatory ventilator; OR: odds ratio; 95% CI: 95% confidence interval; TPN: total parenteral nutrition.

### Clinical features and prognosis

The clinical and laboratorial manifestations seemed more severe in ESBL-GNB than non-ESBL GNB, including higher rate of disseminated intravascular coagulopathy, metabolic acidosis, thrombocytopenia, anemia, prolonged feeding intolerance and higher severity of illness (Table 3). Blood components transfusions and use of inotropic agents were therefore more often required in ESBL-GNB than non-ESBL GNB. All GNB were treated with empiric antibiotics, with no significant difference between groups regarding the antibiotic type administered. However, patients with ESBL-GNB more frequently received inadequate initial empirical antibiotic therapy compared with those with non-ESBL GNB (76.6% vs. 9.5%, *P* < 0.001), and time to adequate antibiotic therapy was also longer (43.4 ± 17.3 hours vs. 3.7 ± 10.4 hours, *P* < 0.001). ESBL-GNB was associated with a poorer outcome than both non-ESBL GNB and the non-infected controls, including a higher rate of infectious complications (20.8% vs. 9.2%, *P* = 0.008) and sepsis-attributable mortality (15.6% vs. 7.9%, p = 0.049).

**Table 3:** Clinical manifestations, treatment and outcomes of extended-spectrum β-lactamase producing Gram-negative bacteremia (ESBL-GNB) compared with non-ESBL GNB.

	ESBL-GNB (n = 77 episodes)	Non-ESBL GNB (n = 316 episodes)	*P* value
Clinical manifestations			
Prolonged feeding intolerance (> 3 days)	44 (57.1)	108 (33.6)	< 0.001
Coagulopathy and/or GI bleeding	31 (40.3)	93 (23.6)	0.076
Disseminated intravascular coagulopathy	21 (15.3)	42 (8.4)	0.005
Septic shock	23 (29.9)	61 (19.3)	0.062
Laboratory characteristics			
Leukopenia (WBC count < 4,000/uL)	18 (23.4)	74 (23.4)	0.983
Leukocytosis (WBC count > 20,000/uL)	28 (36.4)	89 (28.2)	0.167
WBC shift to left (immature WBC ≥ 20% total WBC)	29 (37.7)	75 (23.7)	0.021
Anemia (hemoglobin < 11.0 mg/dL)	49 (63.6)	153 (48.4)	0.022
Thrombocytopenia (platelet < 80,000/uL)	47 (61.0)	148 (46.8)	0.031
C-reactive protein^&^ (mg/dL), median (IQR)	70.5 (29.7–141.5)	63.3 (18.6–121.5)	0.130
Metabolic acidosis requiring jusomin replacement	33 (42.9)	80 (25.3)	0.003
NTISS score at most severe day of bacteremia, mean ± SD	18.0 ± 5.5	16.5 ± 5.0	0.023
Empirical antibiotic treatment			0.865
Combination therapy	73 (94.8)	303 (95.9)	
β-lactam + aminoglycoside	13 (16.9)	48 (15.2)	
β-lactam + 3^rd^ generation cephalosporin	17 (22.1)	59 (18.7)	
Glycopeptide + aminoglycoside	2 (2.6)	12 (3.8)	
Glycopeptide + 3^rd^ generation cephalosporin	27 (35.1)	125 (39.6)	
Glycopeptide + Carbapenem	7 (9.1)	24 (7.8)	
Above combination + anti-anaerobes (metronidazole)	7 (9.1)	35 (11.1)	
Monotherapy	4 (5.2)	13 (4.1)	
3^rd^ generation cephalosporin	2 (2.6)	6 (1.9)	
Carbapenem	2 (2.6)	3 (0.9)	
Glycopeptide	0 (0)	4 (1.3)	
Inadequate antibiotics within 24 hours after bacteremia onset	59 (76.6)	30 (9.5)	< 0.001
Removal of central venous catheters	21/67 (31.3)	84/256 (32.8)	0.884
Requirement of blood transfusion(s)	62 (80.5)	191 (60.4)	0.001
Required intubation/ventilator support with HFOV	36 (46.8)/ 8 (10.4)	125 (39.5)/ 22 (7.0)	0.301/0.310
Outcomes			
Infectious complications*	16 (20.8)	29 (9.2)	0.008
Persistent bacteremia^¶^	7 (9.1)	11 (3.5)	0.071
Sepsis attributable mortality	12 (15.6)	25 (7.9)	0.049

All data were expressed as number (percentage %), unless indicated otherwise; WBC: white blood cell, NTISS: Neonatal Therapeutic Intervention Scoring System, NEC: necrotizing enterocolitis, IQR: interquartile range, HFOV: high frequency oscillatory ventilator.

^&^CRP normal range: < 5 mg/dL.

*Infectious complications were defined as a newly infectious focus or persistent organ dysfunction which occurred within one week and directed related to bacteremia, but not concurrently at onset of bacteremia.

^¶^Persistent bacteremia was defined as 2 or more consecutive positive blood cultures, at least 48 hours apart, during a single sepsis episode.

The in-hospital mortality rates were comparable between neonates with ESBL-GNB and those with non-ESBL GNB, but both were significantly higher than the uninfected controls (both *P* < 0.001, respectively, after Bonferroni adjustment). Although the incidence of ESBL-GNB significantly decreased after early 2005, the clinical features and prognosis did not change over time (a sepsis-attributable mortality rate of 16.3% before 2004 vs. 14.7% after 2005, other comparisons were not shown). However, the ESBL-producing isolates showed a significantly higher antimicrobial resistant rate to gentamicin and amikacin before 2004 (97.7% and 88.4% vs. 58.8% and 38.2%, respectively, both *P* < 0.001) than after 2005.

The results of univariate and multivariate analyses of risk factors potentially associated with in-hospital mortality are summarized in Table 4. Neonates with lower birth weight or gestational age and those with low apgar score at 5 minutes were at significantly higher risk of mortality, so were female infants when compared with male infants. Risk factors for overall mortality included neonates with ESBL-GNB (when compared with uninfected controls) and several underlying chronic conditions. After adjusting for all variables, neonates with lower gestational age (also lower birth weight), underlying secondary pulmonary hypertension with/without cor pulmonale (odds ratio [OR], 7.22; 95% confidence interval: 2.17–24.06; *P* = 0.001) and infectious complications after bacteremia (OR, 6.66; 95% CI: 2.88–15.40; *P* < 0.001) were found to be at independently increased risk for final mortality.

**Table 4:** Risk factors for in-hospital mortality of the 583 study subjects (including 71 patients with ESBL GNB, 289 patients with non-ESBL GNB, and 231 controls) by univariate and multivariate analysis.

Risk factor	Survived, N = 524, n (%)	Died, N = 59, n (%)	*P* value	Multivariate analysis	
				Adjusted OR (95% CI)	*P* value
Gestational age (weeks), median (IQR)	31.0 (28.0–35.0)	27.0 (24.0–34.0)	< 0.001	1.26 (1.03–1.56)*	0.028
Birth body weight (g), median (IQR)	1495.0 (1040.0–2285.0)	956.0 (698.0–1660.0)	< 0.001		
Male sex	302 (57.6)	25 (42.4)	0.038	1.46 (0.73–2.92)	0.283
Outborn	194 (37.0)	15 (25.4)	0.114		
Low apgar score at 5 minutes (≤ 7)	164 (31.3)	33 (55.9)	< 0.001	1.01 (0.46–2.23)	0.978
Study period			0.321		
2001–2004	228 (43.5)	21 (35.6)			
2005–2012	296 (56.5)	38 (64.4)			
Underlying chronic conditions					
Congenital anomalies	20 (3.8)	4 (6.8)	0.266		
Neurological sequelae	49 (9.4)	14 (23.7)	0.003	1.86 (0.81–4.28)	0.142
Cardiovascular disease	18 (3.4)	10 (16.9)	< 0.001	2.53 (0.46–13.94)	0.288
Bronchopulmonary dysplasia	122 (23.3)	29 (49.2)	< 0.001	2.06 (0.88–4.82)	0.094
Pulmonary hypertension	4 (0.8)	9 (15.3)	< 0.001	7.22 (2.17–24.06)	0.001
Gastrointestinal sequelae	25 (4.8)	6 (10.2)	0.074		
Renal disease	5 (1.0)	7 (11.9)	< 0.001	6.40 (0.56–32.89)	0.135
Pathogens			< 0.001		
Controls	222 (42.4)	9 (15.5)			
Neonates with non-ESBL GNB	253 (48.3)	36 (61.0)		1 (references)	
Neonates with ESBL GNB	57 (10.9)	14 (23.7)		1.48 (0.64–3.45)	0.365
Initial inadequate antibiotics	71/310 (22.9)	15/50 (30.0)	0.287		
Infectious complications	22/310 (6.1)	15/50 (30.0)	< 0.001	6.66 (2.88–15.40)	< 0.001

IQR: interquartile range; ESBL-GNB: Extended-spectrum β-lactamase-producing Gram-negative bacteremia; OR: odds ratio, 95% CI: 95% confidence interval.

*Every two weeks of gestational age decrement. Because of the strong correlation between birth weight and gestational age, only the risk factor of gestational age was enrolled into the multivariate analysis.

### Microbiological results

Of the 61 isolates with identifiable ESBLs, SHV-type family accounted for 41 (67.2%) ESBLs, CTX-M for 23 (37.7%), and a TEM-type for 2 isolates (3.3%) (Table 5). Eight isolates produced 2 kinds of ESBLs. We found no significant differences in epidemiological or clinical characteristics by type of ESBL produced (data not shown). Susceptibility results are also shown in Table 5. More than two-third of all isolates showed resistance to aminoglycosides and trimethoprim-sulfamethoxazole, but fluoroquinolone resistance was noted in only 8 (10.4%) isolates. All isolates were considered to be multidrug resistant [30], and 14 (18.2) showed resistance to all β-lactam/β-lactamase inhibitor combination.

**Table 5:** Distribution of β-lactamase genes and major antimicrobial susceptibility pattern of 77 ESBL-producing gram-negative bacteria.

Gene/microorganisms	*E*. *coli* (total n = 16)	*K*. *pneumonia* (total n = 40)	*K*. *oxytoca* (total n = 8)	*Enterob*. spp.* (total n = 13)	Total (total n = 77)
SHV 1	-	7	-	1	8
2	-	1	-	1	2
2A	-	2	1	-	3
11	-	4	-	-	4
12	-	10	1	8	19
CTX M3	2	1	4	1	8
M14	2	-	-	-	2
M27	5	-	-	-	5
M55/M57	1	-	-	-	1
TEM 1	1	-	-	-	1
TEM-1 + SHV-12	-	-	-	1	1
CTX-M3+SHV-1	-	4	-	-	4
CTX-M14+DHA-1	-	-	1	-	1
CTX-M27+CMY-2	2	-	-	-	2
Unidentified	3	11	1	1	16
Isolates period					
2001–2004	8	23	5	7	43
2005–2012	8	17	3	6	34
**Antimicrobial susceptibility patterns**^¶^	**No. (%) of susceptible isolates**				
Ceftriaxone/Cefotaxime	0/16 (0)	0/40 (0)	0/8 (0)	0/13 (0)	0/77 (0)
Ceftazidime	1/16 (6.3)	2/40 (5.0)	3/8 (37.5)	1/13 (7.6)	7/77 (9.1)
Amoxicillin-clavulanate	7/16 (43.8)	34/40 (85.0)	6/8 (75.0)	6/13 (46.2)	53/77 (68.8)
Piperacillin-tazobactam	12/12 (100)	32/36 (88.9)	2/5 (40.0)	6/7 (85.7)	52/60 (86.7)
Imipenem/Meropenem	16/16 (100)	40/40 (100)	8/8 (100)	13/13 (100)	77/77 (100)
Ciprofloxacin	9/16 (56.3)	39/40 (97.5)	8/8 (100)	13/13 (100)	69/77 (89.6)
Amikacin	13/16 (81.3)	11/40 (27.5)	1/8 (12.5)	1/13 (7.6)	26/77 (33.8)
Gentamicin	7/16 (43.8)	7/40 (17.5)	1/8 (12.5)	0/13 (0)	15/77 (19.5)
Aztreonam	0/8 (0)	0/30 (0)	1/6 (16.7)	0/13 (0)	1/57 (1.8)
Flomoxef	13/13 (100)	31/32 (96.9)	5/5 (100)	2/8 (25.0)	51/57 (87.9)
Trimethoprim-sulfamethoxazole	1/6 (16.7)	8/26 (30.8)	1/5 (20.0)	0/12 (0)	10/49 (20.4)

*Including 12 *Enterob*. *cloacae* isolates and one *Enterob*. *aerogenes* isolate.

^¶^Some of the antimicrobial susceptibilities were not performed, and the resistances to cephalosporin were interpreted according to Clinical and Laboratory Standards Institute recommendation.

With regard to clonality, majority of the isolates were clonally unrelated. Five small clusters of clonally related isolates were found by molecular methods. These included 4 SHV-1-producing *K*. *pneumoniae* between June 2009 and December 2009, 3 CTX-M27-producing *E*. *coli* between February 2012 and March 2012, 2 CTX-M27+CMY-2 producing *E*. *coli* between December 2010 and January 2011, 2 SHV-12-producing *K*. *pneumoniae* between February 2006 and December 2006, and 2 CTX-M3-producing *K*. *oxytoca* between August 2002 and September 2002.

## Discussion

Results from this study demonstrated that exposure to third generation cephalosporin within one month before onset of BSIs and presence of underlying renal disease were independent risk factors for acquisition of ESBL-GNB. Although the frequency of occurrence may seem decreasing recently, ESBL-GNB had a higher severity of illness, more severe clinical manifestations, and more frequently required blood transfusions and mechanical support when compared with their antibiotic-susceptible counterparts. Delayed appropriate antibiotics were more frequently encountered in neonates with ESBL-GNB, and ESBL-GNB was therefore associated with a significantly higher rate of infectious complications and sepsis-attributable mortality than non-ESBL GNB.

An appropriate design for investigating risk factors for infection due to antibiotic-resistant organisms should consider more closely the optimization of control group selection and adjusting for confounding caused by time at risk and comorbid illness [31,32]. The preferred control group should be capable of representing the source or base population or underlying cohort. The shortcoming of enrolling only patients with susceptible organisms as the controls is the biased overestimate of relative risk due to exposure to active antibiotics [31], which was reflected in the finding of gentamicin as the independent risk factor when only the antibiotic-susceptible group was compared. In order to eliminate the possible effects of changing practice policies over time, the second controls were randomly selected from the same distinct source (the same NICU at the same period). Furthermore, previous studies investigating risk factors for ESBL-GNB in the NICU were limited by enrolling only very low birth weight infants or the first episode of LOS [5,13,17,18], which may not be equivalent to the whole circumstances of ESBLs. Therefore, our case-control-control design which had been proven useful in previous studies [33,34] seemed more convincing.

Based on our multivariate analysis of case-control-control study design, risk factors for the occurrence of GNB and the emergence of ESBLs can be identified separately. Antibiotic exposure, especially the third generation cephalosporin, was demonstrated to play an important role of developing resistance after selection pressure, but it was the presences of underlying chronic conditions and uses of medical devices that predisposed NICU patients at risk of developing an episode of GNB. The conclusion in this study is more or less similar to our previous study that concluded 3^rd^ generation cephalosporin and underlying renal disease were independent risk factors of multi-drug resistant gram-negative bacteremia [24]. However, the bacterial pathogens were different, and this study did not enroll the more threatening *Pseudomonas* spp. and challenging *Stenotrophomonas maltophilia*.

There have been several studies investigating the relationship between antibiotic use and the emergence of ESBL-producing bacteria [15–18,35], but the effects of different antibiotics or the duration to their use on producing ESBL resistance were rarely addressed [36,37]. Although broad-spectrum antibiotics are associated with emergence of EBSL GNB in this study, further studies are still warranted to evaluate the cumulative effects of antibiotic exposure, the different spectrum and time to prior antibiotic exposure. Therefore, we can have better prediction of drug-resistance bacteria when clinical sepsis is encountered.

In general, bacteria that produce ESBLs to cause antimicrobial resistance are not more virulent than their antibiotic-susceptible counterparts. However, delay in initiating appropriate antibiotics, which is significantly frequently seen in patients with ESBL-GNB, was thought to be an important contributory factor towards final adverse outcomes [38,39]. From this study, we assume that ESBLs significantly cause delay of initiating effective antibiotics, which resulted in progression or persistence of septic symptoms, and then subsequently the occurrence of infectious complications. We found that neither ESBL-GNB nor inappropriate empiric antibiotic was the independent risk factor for mortality. There were other factors that contributed independently to final mortality, such as occurrence of infectious complications or underlying chronic diseases [23,24].

In our NICU, only 10.4% of the ESBL isolates presented resistance to fluoroquinolones, but most (> 70%) were resistant to aminoglycosides and trimethoprim/sulfamethoxazole. These results seem compatible with our antibiotic policies in the NICU, since third-generation cephalosporins and aminoglycosides were significantly more frequent prescribed than quinolones were. Literature review showed that ESBLs from different sources often show different antibiotic susceptibility patterns [3,34,40–42].

A significant decrease in ESBL-GNB was noted since early 2005, which may be associated with the reinforcement of hand hygiene, implementation of alcohol-based handrub and augmentation of aseptic care cover since 2003 to 2004. In our NICU, the nosocomial infection rate was reduced since the end of 2004 [43], which may result in the decrease of antibiotic usage and then the emergence of ESBL-GNB. The molecular data also showed that most ESBL-associated bacteremias were caused by clonally unrelated strains. Several episodes of ESBL-GNB occurred sporadically in a previously healthy patient without any identifiable risk factors, whom might possibly be transmitted from the environment or contaminated hands of healthcare workers [11]. It cannot be overemphasized again that hand hygiene can reduce healthcare-associated infections, including those caused by ESBL-producing bacteria.

In contrast to recent studies that have demonstrated CTX-M enzymes as the predominant ESBLs in all Europe, Canada, South America, and Asia [29,42,44,45], the main enzymes characterized in our NICUs were SHV enzymes, especially SHV-12. However, we noticed an obvious trend between different ESBLs and bacteraemic isolates of *Enterobactericeae* in our NICUs. Taking together, we may assume that CTX-M-type ESBLs are now prevalent in most countries [44,45]. In *Klebsiella* spp. and *Enterobacter* spp., SHV was still the most common ESBL type, but CTX-M-type ESBLs were also present in both organisms.

There are some limitations in this study. Given the extended period of study subjects and retrospective nature, the changing incidence and clinical practices over time can be the biases to our results. Our study population was from a single medical center, and the results may be less generalizable than those from multicenter and prospective observational design. In the outcome analysis, the number of events (ie, deaths) was low, which limited the power of the multivariate analysis. Finally, the scope of molecular epidemiological studies was limited, and a prospective surveillance study should be considered in future investigations.
